# Clinical, Dermoscopic, and Histopathologic Aspects of Amelanotic Lentigo Maligna Melanoma

**DOI:** 10.5826/dpc.1104a137

**Published:** 2021-10-01

**Authors:** Catalin Mihai Popescu, Corina Barna, Alexandru Metea, Razvan Theodor Andrei, Mona Taroi

**Affiliations:** 1 Carol Davila University of Medicine and Pharmacy Bucharest, Dermatology Department, Bucharest, Romania; 2 Military Emergency Hospital Sibiu, Dermatology Department, Sibiu, Romania; 3 Military Emergency Hospital Sibiu, Head and Neck Surgery Department, Sibiu, Romania; 4 Synevo Laboratory Bucharest, Pathology Department, Bucharest, Romania

**Keywords:** amelanotic, lentigo maligna melanoma, dermoscopy

## Introduction

Amelanotic melanomas represent 2% of all melanoma subtypes. Amelanotic lentigo maligna and amelanotic lentigo maligna melanoma are rare entities. Clinical suspicion is very low and the clinical differential diagnosis includes a variety of benign and malignant lesions such as basal cell carcinoma, squamous cell carcinoma, or dermatitis [1].

## Case Presentation

**Figure 1:**
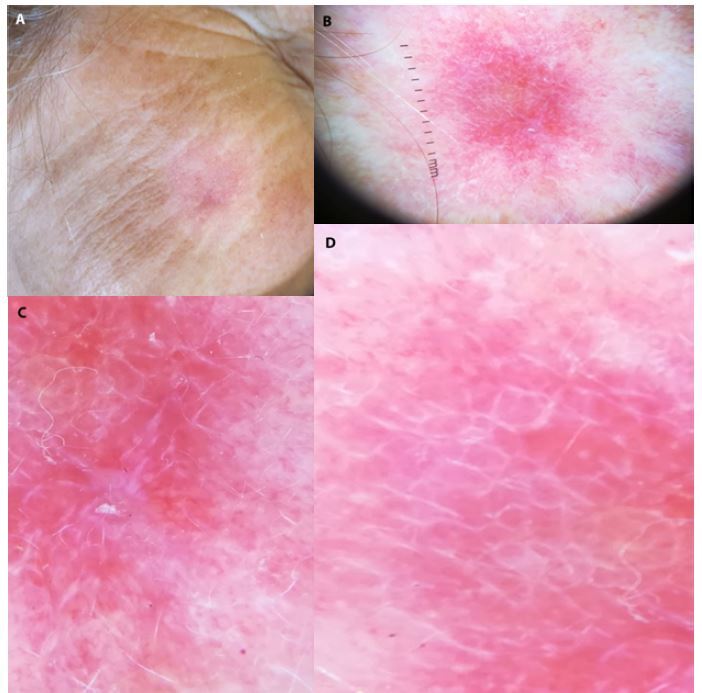
(A) Clinical aspect. Pink plaque with white halo on the right cheek. (B) Polarized dermoscopy of the entire lesion showing milky red areas, perpendicular white lines and polymorphous vessels. (C, D) Polarized dermoscopy (detail) showing perpendicular white lines and polymorphous vessels.

A 73-year-old woman presented with a 4-month history of a pink plaque with a white halo on the right cheek (Figure 1).

**Figure 2:**
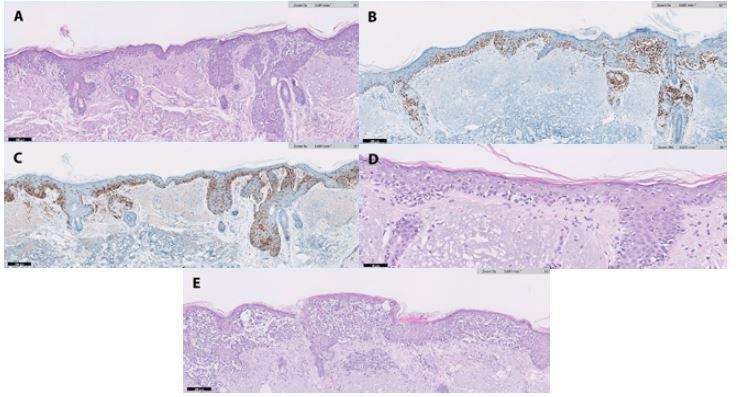
(A) Junctional amelanotic proliferation with a lentiginous pattern of growth, intraepidermal ascending cells, and deep follicular extension (H&E). (B) SOX10 Stain. Junctional amelanotic proliferation with a lentiginous pattern of growth, intraepidermal ascending cells, and deep follicular extension. (C) MelanA Stain. Junctional amelanotic proliferation with a lentiginous pattern of growth, intraepidermal ascending cells, and deep follicular extension. (D) Junctional amelanotic proliferation with a lentiginous pattern of growth (H&E). (E) Irregular nests formation with bridging of rete ridges and small foci of papillary dermal invasion (H&E).

Dermoscopic examination showed polymorphous vessels, milky red areas, and polarizing specific perpendicular white lines (Figure 1, A–D). Punch biopsy revealed a predominantly lentiginous proliferation within the basal layer of epidermis, with forming nests, consisting of epithelioid atypical cells, including areas of thinning of epidermis with loss of rete ridges, and indefinite borders. Severe solar elastosis was present within the dermis. No melanin pigmentation was identified. Tumor was excised and upon complete removal immunohistochemical analysis was carried out. The specimen shows a junctional amelanotic proliferation with a lentiginous pattern of growth (Figure 2A), irregular nests formation with bridging of rete ridges (Figure 2E), intraepidermal ascending cells, deep follicular extension and small foci of papillary dermal invasion (Figure 2E). These features were confirmed by SOX10 stains and MelanA (Figure 2, B and C). Diagnosis of amelanotic lentigo maligna melanoma was made with Breslow thickness 0.7 mm.

## Conclusion

Amelanotic lentigo maligna and amelanotic lentigo maligna melanoma are very rare with very low clinical suspicion and performing dermoscopy on pink lesions can help raise the suspicion of this diagnosis. To our knowledge, even if dermoscopic features of amelanotic melanoma have been described in several articles, there is no evidence reporting on dermoscopic findings of amelanotic lentigo maligna or amelanotic lentigo maligna melanoma.

Polarized dermoscopy allows better appreciation of deeper structures such as collagen and vessels. Perpendicular white lines or shiny white streaks are shiny, bright, often orthogonal, linear streaks seen only with polarized light in dermatofibromas, scars, melanomas, basal cell carcinoma (BCC), and melanocytic naevi, especially Spitz naevi [2]. Dermoscopy is therefore a tool that helps distinguish between inflammatory pink lesions and tumoral pink lesions. Even if they may be present in benign tumors, perpendicular white lines are also a clue to malignancy, although these were more commonly observed in invasive melanomas rather than thin melanomas [2]. Nevertheless, the presence of perpendicular white lines could also represent a guide in the diagnosis of thin melanomas, especially those with few diagnostic criteria, as reported in this situation [2]. In conclusion, the presence of perpendicular white lines should lead to biopsy. In this particular case, the absence of clear-cut clinical and dermoscopic features for an alternative diagnosis was an additional reason for performing biopsy. Diagnosis of amelanotic lentigo maligna melanoma can only be made by histopathology and immunohistochemical stains are very helpful in these cases.
